# Editorials

**Published:** 1890-06

**Authors:** 


### HOMŒOPATHIC PHYSICIAN,

A MONTHLY JOURNAL OF

HOMŒOPATHIC MATERIA MEDICA AND CLINICAL MEDICINE.

If our school ever give up the strict inductive method of Hahnemann, we are lost, and deserve only to be mentioned as a caricature in the history of medicine."—CONSTANTINE HERING.

Vol. X. JUNE, 1890. No. 6.

### EDITORIALS.

THE ARTIFICIAL FEEDING OF INFANTS is one of the most difficult problems with which the physician has to deal. If one could rely upon the various commercial foods with which the market is flooded, the question would at once be solved. Unfortunately, a trial will convince that they are generally not to be trusted. This is the testimony of all physicians whose affirmation is of worth.

The season is now at hand when this subject is of the greatest importance, and the closest attention should be given to it. In a report of a special committee of the American Medical Association, appointed for the purpose of collecting statistics on the matter of infant feeding, the evidence was all in favor of cow's milk in some form, and against the many commercial foods.

All who have had experience will attest the truth of this. No infant should be given artificial food so long as it is possible for the mother to provide the natural, until it has arrived at the proper age for taking other than the mother's milk.

When we arrive at the point where other than natural food is necessary, we should give to it much care and thought.

Taking, then, cow's milk as the best substitute, what preparation is requisite to make it suitable? Many and diverse are the ideas in respect to this. In the following we think we give the best that is known on the subject.

It should be borne in mind that the composition of milk varies considerably, according to the food given the cows. It is a platitude to say that the milk must always be alkaline; and that litmus paper should always be used to test it; not trusting to taste. Lime water and carbonate of soda should never be used to make an acid milk alkaline. Better milk for infant feeding may often be had by taking the cows from the pasture and stall-feeding them. Before being used the milk should stand two or three hours, and then the upper third portion only should be used. This will permit the more solid parts to fall to the bottom, and thus we get rid, in part, of the most indigestible portion—the casein—the curd-forming matter. The water used for diluting should always be boiled, and cooled before adding it to the milk.

From birth to the third week, three parts of water to one part of milk; from the third to the sixth week, two parts of water to one of milk; from the sixth week to the fourteenth week, one-half water; from the fourteenth week to four and one-half months, one-third water; from four and one-half months to the sixth month, one-quarter water. After that age no further dilution is required, unless it is found to disagree. In some cases a small amount of table salt will be required to make it more digestible. It is to be sweetened with sugar of milk.

One of the greatest difficulties to be overcome is the formation of curds. This may be avoided to a great extent by the addition of some farinaceous substance in which the starch has been changed to dextrin. Malt sugar answers here, or, four teaspoonfuls of barley flour, boiled for ten minutes in a pint of water, and then add, when cooled to blood heat, a half to a third of a teaspoonful of liquid extract of malt.

The time may come when we shall have difficulty in finding any food that will agree, and then we shall have to resort to various preparations, in the making of which the main idea must be kept in view—*i.e.*, something readily absorbed, free from starch, and containing the necessary elements of nutrition. Barley water, rice water, oat-meal water, bran tea (a teacupful of wheat bran to a quart of water, boil several minutes, add a little milk, and sweeten to suit). A good food is a teaspoonful of crushed barley, boiled in five to eight ounces of water for twelve or fifteen minutes, with a little salt. Strain through a linen cloth. For a child six to eight months old, equal parts of this and boiled milk. As the child grows older the milk should prevail. Whey is of benefit where there is indigestion. Care must be taken to have all food properly heated before giving.

Infants under three months old should not be fed oftener than once in two and a half hours. Above that age, once in three hours. Some children require larger quantities of food than others. Experience in each case will soon enable one to determine the quantity. Under six weeks of age, about one and a half ounces should be given at each feeding; about two and a half ounces to those three months old ; gradually increasing the quantity each month until the tenth, when about five ounces should be given. Cleanliness of everything used should be insisted upon.

A writer, who has had large experience in an institution where numbers of infants are received says that he has found the best success depends upon the freshness of the milk, and that, by paying attention to this the mortality was reduced from 90 per cent. to 33.5 per cent. He "had not been able to raise a single infant on any of the commercial artificial foods, but children taken to the country where they could get cow's milk fresh, did well and thrived." G. H. C.

---

Surgical Treatment of Uterine Troubles.—Although we should never be willing to trust an enemy, on all occasions we are justified in receiving what we know to be of value, even from our greatest opponent.

If we need any testimony (which we do not) to the efficacy of our application of the law of therapeutics, we need only go to our friends (the enemy) who practice *regular* medicine. Frequently a ray of light seems to penetrate their field of vision, and thus they see what an amount of harm much of their treatment is doing. This occurs only after a long period in which their want of success becomes so glaringly apparent that they are forcibly obliged to stop and give some thought to their lawless and mortal methods.

The followers of Hahnemann never have cause to blush for adhering strictly to his teachings. Depending on no theory, they are not to be confuted with new pathological ideas, and with suppositions regarding the treatment of the sick. It is always found, when any seemingly new scheme regarding disease and its treatment is advanced, that, if it be of any value, Hahnemann and his disciples have been utilizing it for years.

Among the hurtful crazes is the treatment of uterine affections by surgical procedures, and various injurious drugs, both topically and internally. From time to time we read of feeble attempts to call a halt to this treatment, but they seem so infirm that they make little or no impression.

A recent issue of *The Lancet*, of London, has an editorial on this subject, and we think an extract from it will go to prove the truth of what we have said above: "We have from time to time directed attention to the insignificance of certain conditions of the uterus which have been supposed to hold an important place in the pathology of that organ, and have combated pathological theories which are not only wrong, but which also involve methods of treatment that are often dangerous to life and generally injurious to health both of body and mind of the patients concerned. It has been a misfortune to women that from the time—now fifty years ago—when attention became directed to the diseases peculiar to the sex, their study has proceeded along very narrow lines, and pathological theories have been formulated upon insufficient and often erroneous data, and made the basis of universal treatment." The writer then goes on to state that at one time inflammation of the cervix was set down as the base of all troubles in women, and that caustics were applied for months. Then stricture of the cervical canal was the fad, to be treated by dilatation. Flexions and displacements followed this, with the legion of pessaries, which still exist, to the lasting hurt—in most cases—of those who use them. Finally pinhole os, conical cervix, laceration of cervix were and still are blamed with woman's ailments. And such cutting, cauterizing, and drugging which have been applied are enough to terrify the bravest!

To state this is sufficient. Every Hahnemannian can testify to its truth. Any woman who comes to him for relief for symptoms indicative of trouble in the uterine region, and who has been unfortunate enough to have been treated by the scientists (?), brings evidence of her sufferings being due to the treatment received.

But what of those who, professing to know better by taking the name of homœopathist, yet try to feebly imitate those who only hold them in contempt for following their methods?

> "They should hear
> On all sides, from innumerable tongues,
> A dismal, universal hiss, the sound
> Of public scorn."

G. H. C.

---

Blunders.—The article found on another page of this number, entitled "An Awful Blunder," should be so indelibly impressed upon the minds of all who read it that meddlesome midwifery should never even suggest itself to any of them. We recently read of two other cases of complete inversion of the uterus following labor, both of which were due to traction on the cord, and one of which was fatal. In these cases midwives were in attendance. The physicians in the case quoted in full certainly could not have had the dangers of meddlesome midwifery fixed very deeply in mind, else they would not have to bear the burden of having caused their patient's death. Any man is liable to blunder, but to blunder so awfully requires either an ignorance so profound that light could never penetrate it; or a carelessness so gross that it is unpardonable.

We were recently told of the case of a young woman, a primipara, whose lacerated perineum was permitted to take on gangrene until the entire perineum to the anus and beyond was completely destroyed. Her attendant, we were informed, was one who is high in the American Medical Association. Some few years ago we saw, in a village of Central New York, a young man who had been made blind by his doctor (?) ordering fly-blisters to his eyes for a simple ophthalmia. This man was also a regular, and of the ranks of the scientists.

A short time before the death of the late General W. S. Hancock, we saw an article in the New York *Medical Record*, condemning, as hurtful, the use of the knife in carbuncle. Soon after this we read in the same journal an account of the treatment of the case. The knife was used; the General grew worse. Then his physicians (regulars) discovered sugar in the urine. The patient died. The doctors said his death was due to diabetes. Physiologists—particularly, Brown-Sequard—have shown that irritation at the base of the brain will cause sugar in the urine. The carbuncle was in a position to cause more or less irritation of the base of the brain. After the General's death we wrote to the editor of the *Medical Record*, calling his attention to the article condemning the use of the knife, and to the treatment of General Hancock, and to sugar being found in the urine after irritation at the base of the brain. This is his reply: "You are right. We have already noticed the fact in our last issue." He had given the above facts no notice whatever. However, the treatment of that case was not a blunder—it was scientific medicine! G. H. C.

---

THE WARD'S ISLAND "DIFFICULTY" IN NEW YORK.—Under the above heading, our readers will remember that we have given some notices of this bitter controversy, first in the January number, at pages 5 and 32, and again in the April number at page 188.

As a sequence to what has been previously stated, we now commend to the attention of our readers the correspondence of Professor Carleton, which appears in this number at page 272.

The correspondence was originally intended for publication in *The New York Medical Times*, but as its editor, Dr. Egbert Guernsey, has seen fit to omit it from the pages of his journal, it becomes the agreeable duty of THE HOMŒOPATHIC PHYSICIAN to lay it before the profession. All *our* readers will warmly approve the sentiments Professor Carleton has so incisively expressed in his letter, and will easily understand why its publication in *The Times* should be omitted.

The original resolutions against which Dr. Carleton protests are but a disguised repudiation of the homœopathic principle.

This principle, as a law, lifts the healing art to the level of the exact sciences, and it only remains for us to so practice it that we may enrich our materia medica with provings and confirmations by actual clinical experience, and thus securely *establish* Homœopathy in its destined place as an exact science.

To attain this end, all the time and all the energy of the consistent homœopathist are absorbed in the eternal relentless search for the simillimum. There are those who are unwilling to devote themselves to such exacting labor, preferring an easier method of practice. All these delinquents, therefore, seek an escape from the trammels of law. Such an escape they find in these resolutions which place the execution of the law at the doctor's *caprice*, and therefore relegate practical therapeutics to the old ground of theory and speculation (rationalism).

Professor Carleton, therefore, cannot be too highly commended for entering his vigorous protest against such "resolutions" being saddled upon the profession, and we are sure our readers will cordially support him in his stand. W. M. J.

---

ABUSES OF QUININE.—One of our contributors writes to us asking for an article upon the evils of Quinine in massive doses. We appeal to all our subscribers to send us whatever experience they have had with this drug, that we may publish it, and so enable the profession to get a comprehensive idea of what Quinine dosing really means.

